# Supplementary figures and images for: Persistent deleterious effects of a deleterious Wolbachia infection
Source: PLoS Negl Trop Dis. 2020 Apr 3;14(4):e0008204. doi: 10.1371/journal.pntd.0008204 (PMC7159649; doi:10.1371/journal.pntd.0008204)

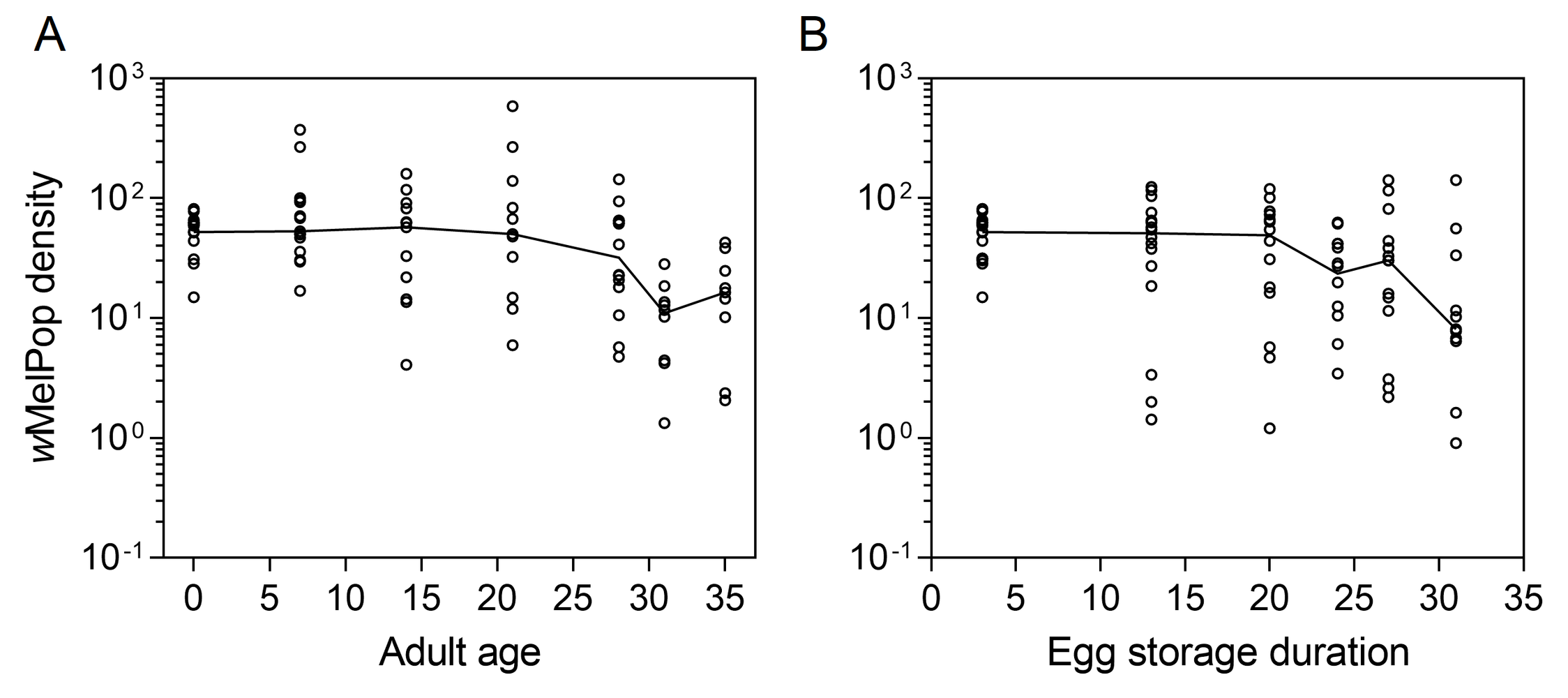

Supplement: S1 Fig — Relative Wolbachia density of wMelPop-infected females with increasing (A) adult age or (B) egg storage duration. Each dot represents the Wolbachia density of a single female, while solid lines join the median densities for each time point. (TIF) [file pntd.0008204.s002.tif]

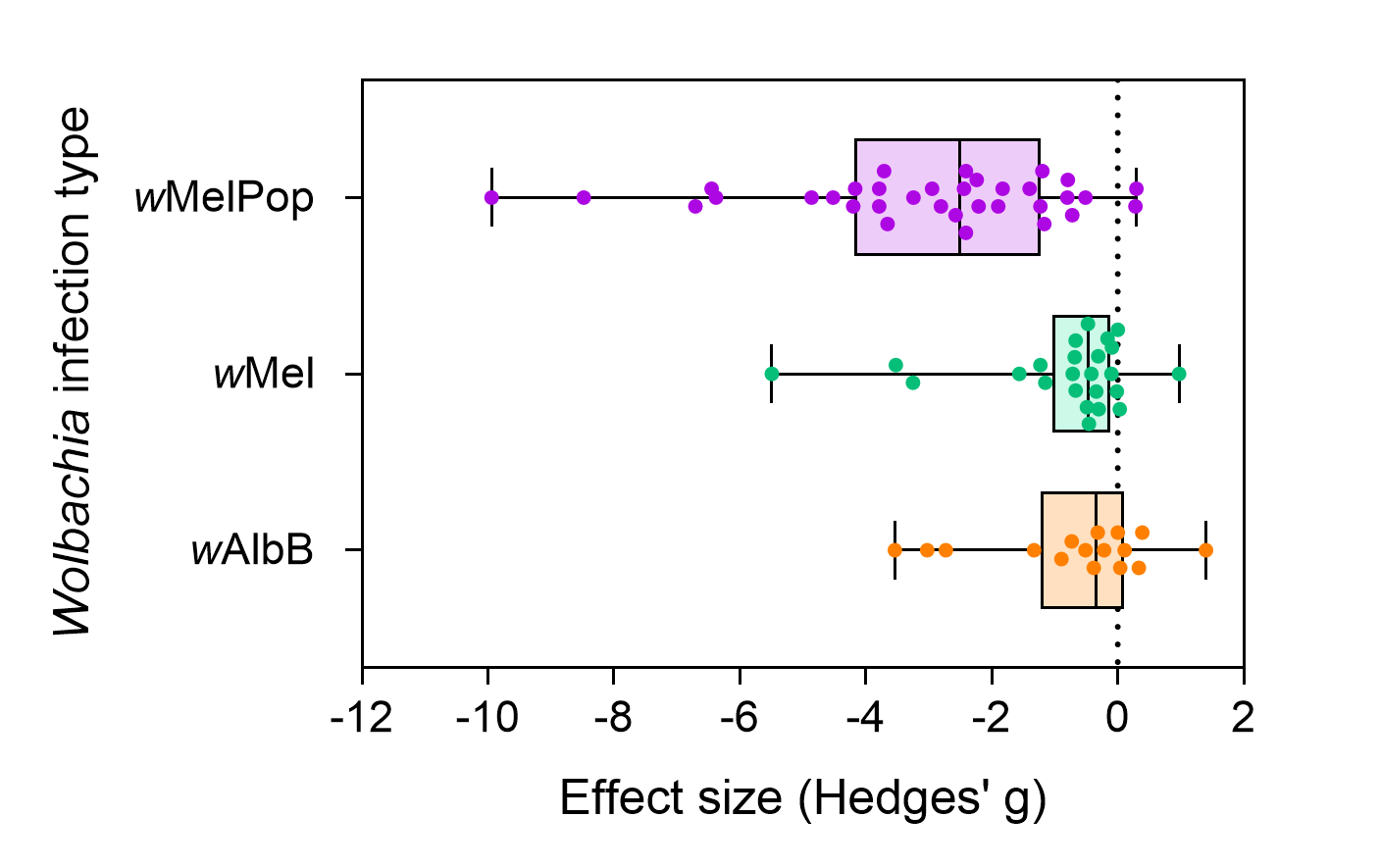

Supplement: S2 Fig — Relative fitness is expressed in terms of effect sizes (Hedges’ g), where values below zero indicate a fitness cost. Each dot represents a single fitness estimate. Box plots show medians and interquartile ranges, with error bars representing minimum and maximum values. (TIF) [file pntd.0008204.s003.tif]
