# Supplementary material for: Persistent deleterious effects of a deleterious Wolbachia infection
Source: PLoS Negl Trop Dis. 2020 Apr 3;14(4):e0008204. doi: 10.1371/journal.pntd.0008204 (PMC7159649; doi:10.1371/journal.pntd.0008204)
Supplement: S1 Appendix — (DOCX) [file pntd.0008204.s004.docx]

**S1 Appendix. Lack of *Wolbachia* transmission through mating in *Drosophila melanogaster* and *D. pandora*.**

**Methods**

To test whether the transmission of *Wolbachia* through mating is specific to *Aedes aegypti*, we performed reciprocal crosses between *Wolbachia*-infected and uninfected *Drosophila*. We used two species carrying different *Wolbachia* infections in our experiments: *w*Mel-infected *Drosophila melanogaster* and *w*PanCI-infected *D. pandora*.

*Drosophila melanogaster* were collected in the Yarra Valley, Victoria, Australia in April 2019. Field-collected females were used to establish isofemale lines. Female parents were screened with diagnostic PCR according to Richardson et al. [1]. We maintained isofemale lines from both *w*Mel-infected and uninfected female parents for use in experiments. *Drosophila pandora* carrying a cytoplasmic incompatibility-inducing *Wolbachia* strain (*w*PanCI) were collected near Lake Placid, Queensland, Australia in May 2011. An uninfected line was generated through tetracycline treatment of the *Wolbachia*-infected line. The *Wolbachia*-infected (*pl+*) and uninfected (pl-) *D. pandora* lines are described in Richardson et al. [1]. All *Drosophila* were maintained on cornmeal media in glass bottles at 25°C in a controlled temperature room before experiments commenced in February 2020.

Adults emerging from pupae were collected from bottles within 3 hr of emergence and the sexes were separated under CO_2_ to ensure that females had not mated. We established reciprocal crosses between *Wolbachia*-infected and uninfected flies (Table 1) by aspirating 20 females and 20 males from each *Wolbachia* infection type combination into *Drosophila* vials. We performed crosses to test for both transmission of *Wolbachia* from males to females and from females to males. We also included control crosses where both sexes were either *Wolbachia*-infected or uninfected. Adults were left to mate for 72 hr, after which both sexes were stored separately in 1.7 mL tubes of 100% ethanol.

We tested nine females from each cross for *Wolbachia* presence and density following the same procedure from the main paper (see the “*Wolbachia screening*” section). Nine males from the *Wolbachia*-infected female and uninfected male cross were also included to test for transmission of *Wolbachia* from females to males. We used *w1* primers to amplify *Wolbachia* DNA from both species and used *Drosophila* universal (*RpL40*) primers (see Richardson et al. [2]) as a control (all samples were expected to have robust and similar amplification). Relative *Wolbachia* densities were determined by subtracting the *w1* Cp from the *Rpl40* Cp and then transforming this value by 2^n^. Each sample was tested three times with *w1* and *Rpl40* primers so relative *Wolbachia* densities represented the average of three measurements.

**Results**

All females from the *Wolbachia*-infected and uninfected control crosses were positive and negative for *Wolbachia* respectively, confirming the *Wolbachia* infection status of each line (Table 1). In crosses between uninfected females and *Wolbachia*-infected males, all females were negative for *Wolbachia* (lacking Cp values for the *w1* primer). Similarly, males from crosses between *Wolbachia*-infected females and uninfected males were negative for *Wolbachia*. We therefore find no evidence to suggest that *Wolbachia* can be transferred through mating in *Drosophila*, in contrast to *Ae. aegypti* where this occurs with the *w*Mel and *w*MelPop infections.

**Table 1. Detection of *Wolbachia* in *Wolbachia*-infected and uninfected *Drosophila* following reciprocal crosses.**

| ***Drosophila* species** | **Female infection type** | **Male infection type** | **Sex tested** | **Proportion *Wolbachia*-positive*** | **Median relative *Wolbachia* density (lower, upper 95% confidence interval)*** |
| --- | --- | --- | --- | --- | --- |
| *Drosophila melanogaster* | *w*Mel | *w*Mel | Female | 1 | 2.54 (1.38, 4.65) |
|  | *w*Mel | Uninfected | Female | 1 | 2.21 (1.48, 3.29) |
|  | Uninfected | *w*Mel | Female | 0 | - |
|  | Uninfected | Uninfected | Female | 0 | - |
|  | *w*Mel | Uninfected | Male | 0 | - |
| *Drosophila pandora* | *w*Pan CI | *w*PanCI | Female | 1 | 1.31 (0.94, 1.82) |
|  | *w*PanCI | Uninfected | Female | 1 | 1.54 (1.11, 2.12) |
|  | Uninfected | *w*PanCI | Female | 0 | - |
|  | Uninfected | Uninfected | Female | 0 | - |
|  | *w*PanCI | Uninfected | Male | 0 | - |

*n = 9 for each cross.

**References**

1. Richardson KM, Schiffer M, Griffin PC, Lee SF, Hoffmann AA. Tropical *Drosophila pandora* carry *Wolbachia* infections causing cytoplasmic incompatibility or male killing. Evolution. 2016;70(8):1791-1802. doi: 10.1111/evo.12981

2. Richardson KM, Griffin PC, Lee SF, Ross PA, Endersby-Harshman NM, Schiffer M, Hoffmann AA. A Wolbachia infection from Drosophila that causes cytoplasmic incompatibility despite low prevalence and densities in males. Heredity. 2018;122:428-440. doi:10.1038/s41437-018-0133-7
