# Supplementary material for: Persistent deleterious effects of a deleterious Wolbachia infection
Source: PLoS Negl Trop Dis. 2020 Apr 3;14(4):e0008204. doi: 10.1371/journal.pntd.0008204 (PMC7159649; doi:10.1371/journal.pntd.0008204)
Supplement: Attachment — Submitted filename: response to reviewers final.docx [file pntd.0008204.s005.docx]

Reviewer's Responses to Questions

First, the strain described in this paper is not the Drosophila wMelPop, but a very particular wMelPop variant: wMelPop-PGYP (Woolfit et al. (2013) Genome Biol. Evol.). This variant differs from the Drosophila wMelPop is several ways, the most striking being the deletion of the Octomom genomic region, responsible for wMelPop virulence in Drosophila (Chrostek and Teixeira (2015) PLoS Biol.). This region, present in one copy in wMel and wMelCS, is amplified in wMelPop, while wMelPop-PGYP has zero copies of this region (Woolfit et al. (2013) Genome Biol. Evol.).
 **Thanks for bringing this to our attention. We now clarify in the ‘mosquito strains and colony maintenance’ section that the strain described in the paper is the wMelPop-PGYP variant and we also mention this point in the introduction (line 60-61). We also note in the discussion that the lack of genomic changes since cell line adaptation (at least after four years in the mosquito) demonstrated in Woolfit et al. is somewhat consistent with the results here and highlight in the discussion the difference with Drosophila (lines 449-451). We have also clarified our terminology around wMelPop-PGYP vs wMelPop in the introduction and methods and elsewhere where appropriate.**

Lines 280-281 – wMelPop-CLA, the variant passaged through mosquito cell lines, which was introduced into the Aedes aegypti mosquitoes (from mosquito cell lines), already replicates much less than wMelPop in Drosophila melanogaster (McMeniman et al. (2008). Appl. Environ. Microbiol.). It is also a null for Octomom and seems like a more fair comparison for the wMelPop-PGYP assayed here.

**We agree that this is a better comparison, but Wolbachia density still increases with age in this study. We now refer to wMelPop-CLA and McMeniman et al. 2008 in this section of the results (lines 297-8). We also refer to McMeniman et al. 2008 and Woolfit et al at (lines 465-467) in a revised paragraph in the discussion and we now also refer to Octomom in the discussion (lines 449-451).**

Line 300 – I would replace “fecundity” with “egg production”.

**Done**

Line 112-113 – What was the age of females blood-fed for colony maintenance? wMelPop variants causes a range of pathologies late in life, but young insects seem to be normal.

**We now state that females were fed approximately 1 week after adult emergence. We also state when eggs were normally hatched post-collection (although this varied due to weekends / holidays, etc.)**

Figure 4 - The loss of wMelPop from populations crossed to uninfected males seems very fast. Could 2% of transmission failure and ~20% fewer eggs explain an almost complete Wolbachia loss over 4 generations? More detailed method description would help to understand this result – eg. what was the age of the females when they were crossed to uninfected males? Were the eggs stored before hatching to provide advantage to the uninfected individuals? Does the model described in lines 461-470 predict the loss of infection under these circumstances?

**We now include more detailed methods for this section, including the age when adults were crossed, blood fed, etc.. These methods would have minimized (but not eliminated) the fitness costs of wMelPop, but we would still expect to see rapid loss in this scenario if there is maternal transmission failure, because there is no cytoplasmic incompatibility occurring.

When wMelPop males and females are crossed together (where there is the potential for cytoplasmic incompatibility if wMelPop starts to drop out), we didn’t see any loss, at least in this experiment. The model in the discussion predicts the loss of wMelPop when cytoplasmic incompatibility is absent, but not when there is near-perfect cytoplasmic incompatibility.**

Reviewer #2: Line 128 Improper italics on Alb. Also line 130. Check throughout manuscript.
Line 132-138 Belongs in the results not methods. Reader will be confused/skeptical until they get the full explanation.

**We have italicized *wAlbB* fully here because we are referring to the name of the primers, not the *w*AlbB strain. Because we use this weak and strong designation throughout the results (and before the mating transmission section), we feel that it is important to define this in the methods section.**

The paper would have been strengthened by the addition of a wMelPop line from a different laboratory, although this may not have been possible.  The generality of the conclusions is questionable given that it is only one line/ one colony effectively.  Although the experiments are well designed to maximise sample size within that one colony, it is still one colony.  The colony was also collected from the field, which may have imposed very strong selection on the original stock.  It would have been interesting to compare with a colony that has not left the lab.

**Unfortunately we didn’t have access to a line that has never left the lab, but we agree that this would have been a worthwhile comparison. We now include a line in the discussion explaining that the short period in the field is likely to have imposed strong selection for attenuation (line 467-468).**

Specific comments:

Line 109:  by North Queensland material, do the authors mean Wolbachia-uninfected material? Presumably but please specify as North Queensland material can also mean Wolbachia infected.

**We now specify that the North Queensland material was uninfected in line 115.**

Methods – please also provide detail on rearing of larvae

**We now include details on the rearing of larvae in lines 117-119.**

Line 120 – what does this mean? Over how many years? Which experiments were done in which years?

**We now specify that experiments were performed over seven years. Although most experiments were performed in 2019, some of the sexual transmission experiments were performed in 2016 while the routine scoring of egg hatch went from 2012 to 2018. We now include this information in lines 125-127.**


Line 128 – please give primers for the wAlb

**We now include a list of all the primers used in a supplementary table (Table S1) and refer to this table in line 141.**

Line 151 – it would be easier for the reader if the authors denoted wMelPop with + and wMelPop negative populations with a minus

**We tried this but the positive and negative signs are not easy to see so we would prefer to stick to the way of referencing strains as in the original version (ie writing out “negative” in full) but we have added a hyphen between wMelPop and “negative” to make it easier to read.**

Reviewer #1: The results are clearly presented, I have just a single suggestion in this department:
Fig. 2C – “incompatible females” on the axis is misleading. How about “Females with 0 viable progeny” or “infertile females”? No CI-defining pathologies were scored here, and the incompatibility of these females is a hypothesis.

**We agree that this may be misleading; we have changed the axis label to “proportion of females with no viable progeny”, consistent with the figure legend.**

Reviewer #2: On line 271 are these females or a mix of M and F?  Hard to know how this compares to the next few lines. Put the data on the same footing.

**We now clarify that wMelPop previously shortened the lifespan of both males and females by around 50%.**

Line 288. Same as above. What does ‘severely mean’? Do you have a number to compare to the next sentence?

**We have decided to remove ‘severely’ since the cost is difficult to quantify (as it changes with female / egg age).**

Reviewer #3: Line 269 – this is fine but it is confounded by the fact that wMelPop mosquitoes were collected from the field, which itself may have resulted in perhaps lower densities or selection in the field for mosquitoes which are better able to tolerate wMelPop infection; it would have been useful if the authors had also been able to access the original laboratory colony?; at the very least the field origin should be discussed

**Unfortunately we don’t have access to the original wMelPop colony, but it would have been useful to include. We now mention in the discussion (lines 467-468) that the short period in the field may have induced strong selective pressures which could explain the attenuation.**

Line 319 – what is meant by population effect here? What exactly is the population? Line 323 – could the authors clarify if experimenter is person that the mosquitoes are feeding on?

**We now define both terms in the methods section, lines 214 and 215 respectively.**

Figure 2: I cannot see the data for uninfected females (2019) in A and B panels - why is this missing?

**It's missing because we did not include the uninfected population in this experiment due to logistical constraints. We state this in the “Re-evaluation of deleterious effects” section, but we now also clarify that the uninfected population was not included under the “Fertility” subheading in the methods section (line 177).**

Line 426 – limited, rather than “no” attenuation. Lifespan shortening phenotype, differences in feeding behavior, and number of eggs per female seem to have changed since 2009, improving the outcomes for infected colonies.

**We have removed the “no”.**

Lines 436-438 – Duplication in Octomom is easy to reverse, but it is not the cause of virulence of wMelPop-PGYP1.

**Thanks for pointing this out: we have reworked this paragraph and removed this sentence from the discussion.**

Lines 493-494 – or heads or legs?

**Good point: it should also be feasible to screen the heads to eliminate false positives. However, screening legs may not work for some Wolbachia strains that are more limited in their tissue distribution. We now suggest that Wolbachia frequencies could be determined from dissected heads.**

The last paragraph contains a mix of vector control ideas, including CI-based suppression and population replacement and subsequent crashing. To make this speculation more complete, assessment of the current susceptibility of wMelPop-PGYP to viruses could be proposed. Also, it is difficult to miss the fact that if wMelPop variants are already difficult to maintain in laboratory mosquito colonies, they are likely to be difficult to deploy in the field.

**It would be interesting to see if the virus-blocking ability of wMelPop has been changed, but we don’t propose releasing wMelPop for permanent population replacement as there are more viable options.
We agree that releasing wMelPop into the field is more challenging than other strains, but it is still feasible to deploy on a large scale, especially if the issues discussed here are taken into account.**


Reviewer #2: Line 501 What do you mean? “deleterious effects and monitoring issues”

**We have clarified what we mean by monitoring issues (false positive detections due to transmission of *Wolbachia* through mating). We now also specify deleterious effects due to infection (with *w*MelPop); we hope this clarifies things.**

Line 505 Explain why "isolated areas"? Do you mean because there won't be invasion from the outside by Wolbachia free? I think to discuss this properly you need rehash the failed releases a bit - what did the failures look like.  Possibly better in the discussion.

**We now clarify that isolated areas are not easily invaded by uninfected mosquitoes and have added a few more details (see lines 532-534).**

Discussion at lines 472-475:  mating-based transmission would surely lead to significant rates of false positive identification only if there was a large proportion of wMelPop uninfected females already in the population, therefore the underlying reason for loss must lie elsewhere.

**Sorry for the confusion; we agree that the loss of wMelPop is because of maternal transmission failure, though mating-based transmission will make it harder to detect this loss. Since we discuss this issue in the following paragraph we have removed the line about mating transmission from this section.**

Discussion at line 482: this appears to be a phenomenon restricted to wMelPop and wMel in this particular study, not wAlbB; has this been observed in Drosophila lines infected with wMelPop?; it is an important observation so would it be feasible for the authors to perform additional experiments with Drosophila flies infected with wMel and wMelPop?

**We don’t have a wMelPop line, but we have now tested this with wMel in *Drosophila melanogaster* and *w*Pan CI in *Drosophila pandora* by performing reciprocal crosses similar to the mosquito experiments. We did not find any evidence for transmission through mating in either species. We have included the results of this experiment in a supplementary appendix (Appendix S1).**

Further point on the Wolbachia mating transmission experiments: females that have wMePop transferred via seminal fluid would be weakly positive in qPCR of whole bodies, as the abdomen only qPCRs are close to Cq of 29 (line 394).  Generally values close to 30 in qPCR should be suspect (for any pathogen) and require repetition.  Therefore, it should be possible to screen out weakly positive females from a laboratory line.

**We now clarify that it should be possible to identify weakly positive (false positive) females in a laboratory setting.**

Line 493: unless adults are held singly post hatching from the field it seems somewhat difficult to implement this recommendation of only screening unmated adults

**Testing unmated adults is relatively straightforward for two reasons: sexing pupae is quick and can be done by eye (although may not be 100% accurate for field-collected samples), and adults will not mate for at least 24 hr, post-emergence. If newly-emerged adults are collected twice daily (or daily), even without pupal sexing, it is possible to screen unmated adults in large numbers.**

I am not entirely convinced of the author's premise that rearing in the laboratory should have selected for less virulent Wolbachia.  Life shortening does not kick in until late in life. Most labs, knowing the line is sensitive, rear it carefully.  That means taking only the first or second gonotrophic cycle (well before aging or fitness effects kick in) and and not leaving eggs dried down for too long. I think the authors need to give some room for this explanation as to why the virulence has not changed. 

**We now provide more information in the methods about how the mosquitoes were maintained (e.g. lines 117-119, 120-122). We agree that this rearing schedule should avoid most selection (because the large deleterious effects haven’t kicked in), although wMelPop clearly has fitness costs even under these benign conditions. We now explain in the discussion that this rearing schedule may reduce (but not eliminate) selection for attenuation (see lines 457-459).**

Additionally, to make this more than an intellectual exercise, the authors need to do a better job of explaining how, despite failure to spread in Vietnam and Australia, that the wMelPop strain could still be used in the field. Expand/explain.  Also in the discussion. And what about temperature (26 in the lab vs much hotter in the field). This strain may be particularly affected.

**We now explain that the release of both sexes in the field can result in cytoplasmic incompatibility and fitness costs which may suppress populations (once wMelPop has reached a high frequency) (see lines 536-538). In terms of temperature effects, previous work from our lab shows that wMelPop is equally susceptible as wMel to high temperatures, so this issue isn’t specific to wMelPop. Although high temperatures can affect invasion success, wMel can persist in most tropical release sites despite this vulnerability as we have recently demonstrated (Ross, Axford et al. 2020 PloS Negl Trop Dis. Heatwaves cause fluctuations in *w*Mel *Wolbachia* densities in Aedes aegypti mosquitoes).**

Reviewer #3: In general the paper is well written and the studies are adequate. I have two principal concerns as discussed above:

1) we are seeing patterns largely restricted to one colony;

**This is a reasonable concern, but one that we are unable to avoid given that we couldn’t obtain wMelPop lines from elsewhere. Although we can demonstrate conclusively that some deleterious effects have persisted, our inability to perform direct comparisons limits our conclusions somewhat, which we have tried to not overstate.**

2) one of the most interesting interesting findings - of male to female transmission of wMel and wMelPop during mating - isn't explored further through additional experiments; is this specific to Aedes aegypti or does it occur in Drosophila? what happens to Wolbachia in the spermathecae?  there is no dissection of spermathecae to look at viability or what happens to the bacteria over time post mating. 

**This experiment was carried out to understand why we didn’t detect the loss of wMelPop while it was dropping out of our laboratory colonies. Our concerns were practical, so we did not investigate the mechanism in the current study. This would require several additional experiments which is beyond the scope of this paper. We had a few Drosophila lines available at the time of receiving these reviews, so we have now performed similar experiments in two Drosophila species. The results are available in the supplementary information (see Appendix S1).**

I should note that wMelPop deleterious effects are also observed after many, many generations in Drosophila. This isn't really discussed anywhere.

**Thanks for bringing this up. We now note that deleterious effects are still apparent in Drosophila melanogaster in lines 89-90 in the introduction and refer to Chrostek and Teixeira 2015 (a relatively recent study conducted long after the laboratory line of wMelPop originated) which demonstrates this clearly.**

The title suggests wMelPop is unstable - yet it seems very stable in general over the decade being investigated, except for one particular instance when the infection was lost.  Therefore it is unclear what the authors mean by this.

**We were referring to instability in terms of its occasional loss from laboratory populations. However, since this occurs rarely we have now removed “unstable” from the title.**
